# The Epidemiology of Depressive Symptoms and Poor Sleep: Findings from the English Longitudinal Study of Ageing (ELSA)

**DOI:** 10.1007/s12529-017-9703-y

**Published:** 2017-12-04

**Authors:** Lydia Poole, Marta Jackowska

**Affiliations:** 1 Department of Behavioural Science and Health, University College London, 1-19 Torrington Place, London, WC1E 6BT UK; 2 Department of Psychology, Whitelands College, University of Roehampton, Holybourne Avenue, London, SW15 4JD UK

**Keywords:** Depressive symptoms, Sleep complaints, Health behaviour, Physical illness, C-reactive protein, Fibrinogen

## Abstract

**Purpose:**

The reasons for the comorbidity between depressed mood and poor sleep are not well understood.

**Method:**

Participants were 5172 adults aged 50 years and older from the English Longitudinal Study of Ageing. Sleep was measured via self-report and depressive symptoms using the Centre for Epidemiological Studies Depression scale.

**Results:**

Greater depressive symptoms and sleep complaints were associated with female sex, non-cohabitation, relative poverty, smoking, infrequent physical activity, infrequent alcohol consumption, higher body mass index (BMI), diagnosis of hypertension, coronary heart disease, diabetes/high blood glucose, pulmonary disease, arthritis, and higher levels of fibrinogen and C-reactive protein (all *p* < 0.05). At a 4-year follow-up, depressive symptoms and sleep complaints were both predicted by baseline depressive symptoms and sleep complaints, relative poverty, smoking, physical inactivity, BMI, and arthritis (all *p* < 0.05).

**Conclusion:**

Depressive symptoms and sleep complaints share a range of correlates cross-sectionally and prospectively. These findings highlight the common comorbidity between depressive symptoms and sleep complaints underscoring the need for further research to understand their combined detrimental effect on long-term health and wellbeing.

## Introduction

Depression is now one of the leading causes of global disease burden [1] and subclinical levels of depression in community-dwelling adults are known to be highly prevalent in England [2]. Elevated depressive symptoms have been associated with an array of poor physical health outcomes, including increased risk of diabetes [3] and coronary heart disease [4]. Late-life depression has been associated with dementia [5] and there is some evidence for an association with cancer [6], though this literature is mixed [7]. Understanding why depressive symptoms are adversely related to health is therefore of great importance.

The high level of symptom overlap between depression and physical illness has been proposed as a potential reason for the apparent association. Such hypotheses imply residual confounding may exist, though this explanation overlooks the potential role of somatic correlates of depression. One such example is poor sleep [8–10].

The co-occurrence of depression and sleep symptoms has long been recognised [11] though the temporal sequence has been difficult to establish. Depression is frequently accompanied by adverse changes to sleep, such as problems with sleep onset, sleep maintenance, and early morning awakenings [10]; these self-reported findings have also been confirmed using objective readings of sleep collected via actigraphy and polysomnography [9]. However, despite sleep disturbance being a diagnostic characteristic of major depression, we also know that depression is highly prevalent in those with insomnia [12] and that insomnia sufferers are at increased risk of developing a new-onset depressive episode compared to those without sleep problems [13] and such results have been supported using meta-analysis [14, 15]. Adults sleeping short sleep hours, typically 5 h or less per night, are also at an increased risk of depression [16]. Abnormal sleep in turn is associated with poor health outcomes and an array of physical illnesses [17, 18]. Some authors have urged caution in interpreting the longitudinal associations suggesting instead that sleep disturbance is a prodromal symptom of depression [19], highlighting the need for further understanding of this issue.

The reason why depression and sleep symptoms co-occur is not well understood. We attempted to address this question by examining the participant profiles of adults taking part in the English Longitudinal Study of Ageing (ELSA). These were exploratory analyses which aimed to shed light on the sociodemographic, behavioural, clinical, and biological correlates of depressive symptoms and poor sleep. We also aimed to elucidate if depressive symptoms and poor sleep complaints measured at a 4-year follow-up would be predicted by similar factors. Finally, we aimed to assess the interaction between depressive symptoms and sleep complaints for predicting these same symptoms over time.

## Methods

### Sample and Study Design

Data were taken from ELSA, a nationally representative general population study of adults aged 50 years and older living in England. Further details can be found elsewhere [20]. The sample is followed up every 2 years from 2002 onwards, with refresher samples being added at waves 3, 4, and 6. At every wave, participants complete a computer-assisted personal interview plus a self-completion questionnaire. On alternate waves, a nurse visit is conducted to allow for the collection of blood samples and objective assessments of physical function, such as body mass index (BMI).

This current paper reports data spanning 4 years, from wave 4 (2008/9) through to wave 6 (2012/2013), of core members. Wave 4 and wave 6 were selected since these are the only points at which sleep data were collected. Analysis was performed on a sample of 5172 participants (65.71 ± 8.76 years; 54.6% female) who provided data on all variables, out of a total available sample of 7744 core members. Missing data on hs-CRP (*n* = 1346), fibrinogen (*n* = 1428), and BMI (*n* = 324) means analyses using these variables were performed on a reduced sample size, as indicated in Tables 1, 2, and 3.

**Table 1:** Demographic, clinical and biological characteristics of the sample (*N* = 5172)

Characteristic	Mean ± SD or *N* (%)
Baseline sociodemographics	
Age (years)	65.71 ± 8.76
Female	2824 (54.6)
Ethnicity—White	5088 (98.4)
Married/cohabiting	3740 (72.3)
Net financial wealth—quintiles	
1	718 (13.9)
2	975 (18.9)
3	1060 (20.5)
4	1161 (22.4)
5	1258 (24.3)
Baseline health behaviours	
Current smoker	630 (12.2)
Moderate/vigorous physical activity ≥ 1 a week	1608 (31.1)
Alcohol consumption	
> 5 times per week	1205 (23.3)
1–4 times per week	2073 (40.1)
1–2 times a month/every 2 months (occasionally)	944 (18.3)
Twice a year/never (rarely/never)	950 (18.4)
Body mass index (kg/m^2^)*	28.35 ± 5.20
Baseline clinical factors	
Hypertension	2820 (45.5)
CHD	477 (9.2)
Stroke	178 (3.4)
Diabetes/high blood glucose	486 (9.4)
Cancer	238 (4.6)
Pulmonary disease	208 (4.0)
Arthritis	1922 (37.2)
Baseline biomarkers	
Fibrinogen (g/l)**	3.33 ± 0.52
hs-CRP (mg/l)***	3.02 ± 3.53
Baseline and follow-up mood and sleep problems	
Wave 4 CES-D	0.90 ± 1.56
Wave 6 CES-D	0.90 ± 1.55
Wave 4 sleep complaints	1584 (30.6)
Wave 6 sleep complaints	1626 (31.4)

*N = 4848

**N = 3744

***N = 3826

**Table 2:** Baseline associations between depressive symptoms and sleep complaints and sociodemographic, behavioural and clinical variables (*N* = 5172)

	Depressive symptoms			Sleep complaints			
	Mean (SD)	Test statistic	*p*	Mean ± SD or *N* (%)		Test statistic	*p*
				Low (*n* = 3588)	High (*n* = 1584)		
Sociodemographics							
Age	–	*r* = 0.048	0.001	65.72 ± 8.69	65.68 ± 8.93	*t* = 0.165	0.869
Sex							
Male	0.67 ± 1.35	*t* = − 9.869	< 0.001	1797 (50.1)	551 (34.8)	*χ* ^2^ = 103.750	< 0.001
Female	1.10 ± 1.69			1791 (49.9)	1033 (65.2)		
Ethnicity							
White	0.90 ± 1.56	*t =* − 2.335	0.020	3534 (98.5)	1554 (98.1)	*χ* ^2^ = 1.040	0.340
Non-White	1.30 ± 1.83			54 (1.5)	30 (1.9)		
Cohabiting							
No	1.47 ± 1.90	*t* = − 16.570	< 0.001	916 (25.5)	516 (32.6)	χ^2^ = 27.250	< 0.001
Yes	0.69 ± 1.35			2672 (74.5)	1068 (67.4)		
Wealth							
1 (lowest)	1.64 ± 2.03	*F* = 70.128	< 0.001	386 (10.8)	332 (21.0)	*χ* ^2^ = 167.084	< 0.001
2	1.10 ± 1.70			618 (17.2)	357 (22.5)		
3	0.87 ± 1.48			728 (20.3)	332 (21.0)		
4	0.70 ± 1.38			862 (24.0)	299 (18.9)		
5 (highest)	0.54 ± 1.15			994 (27.7)	264 (16.7)		
Health behaviours							
Smoker							
No	0.82 ± 1.47	*t* = 10.142	< 0.001	3195 (89.0)	1347 (85.0)	*χ* ^2^ = 16.510	< 0.001
Yes	1.49 ± 2.03			393 (11.0)	237 (15.0)		
Physical activity							
< 1 session/week	1.04 ± 1.66	*t* = 9.757	< 0.001	2325 (64.8)	1239 (78.2)	*χ* ^2^ = 92.379	< 0.001
≥ 1 session/week	0.59 ± 1.27			1263 (35.2)	345 (21.8)		
Alcohol							
> 5 times/week	0.66 ± 1.35	*F* = 61.563	< 0.001	897 (25.0)	308 (19.4)	*χ* ^2^ = 90.567	< 0.001
1–4 times/week	0.75 ± 1.41			1506 (42.0)	567 (35.8)		
Occasionally	0.96 ± 1.60			640 (17.8)	304 (19.2)		
Rarely/never	1.48 ± 1.92			545 (15.2)	405 (25.6)		
BMI*	–	*r* = 0.068	< 0.001	28.04 ± 4.92	29.07 ± 5.72	*t* = − 6.413	< 0.001
Clinical factors							
Hypertension							
No	0.81 ± 1.48	*t* = −4.022	< 0.001	1668 (46.5)	684 (43.2)	*χ* ^2^ = 4.845	0.029
Yes	0.98 ± 1.62			1920 (53.5)	900 (56.8)		
CHD							
No	0.85 ± 1.52	*t* = − 7.335	< 0.001	3309 (92.2)	1386 (87.5)	*χ* ^2^ = 29.292	< 0.001
Yes	1.40 ± 1.87			279 (7.8)	198 (12.5)		
Stroke							
No	0.89 ± 1.56	*t* = − 2.016	0.044	3476 (96.9)	1518 (95.8)	*χ* ^2^ = 3.612	0.068
Yes	1.13 ± 1.55			112 (3.1)	66 (4.2)		
Diabetes/high blood glucose							
No	0.87 ± 1.53	*t* = − 4.957	< 0.001	3287 (91.6)	1399 (88.3)	*χ* ^2^ = 13.973	< 0.001
Yes	1.24 ± 1.78			301 (8.4)	185 (11.7)		
Cancer							
No	0.89 ± 1.55	*t* = − 4.022	< 0.001	3425 (95.5)	1509 (95.3)	*χ* ^2^ = 0.092	0.744
Yes	1.14 ± 1.76			163 (4.5)	75 (4.7)		
Pulmonary disease							
No	0.87 ± 1.52	*t* = − 7.937	< 0.001	3472 (96.8)	1492 (94.2)	*χ* ^2^ = 18.878	< 0.001
Yes	1.74 ± 2.13			116 (3.2)	92 (5.8)		
Arthritis							
No	0.69 ± 1.36	*t* = − 13.112	< 0.001	2477 (69.0)	773 (48.8)	*χ* ^2^ = 192.683	< 0.001
Yes	1.27 ± 1.79			1111 (31.0)	811 (51.2)		
Fibrinogen**	–	*r* = 0.085	< 0.001	3.32 ± 0.52	3.38 ± 0.52	*t* = − 3.269	0.001
hs-CRP ln***	–	*r* = 0.084	< 0.001	0.52 ± 1.06	0.71 ± 1.01	*t* = − 4.939	< 0.001

*N = 4848

**N = 3744

***N = 3826

**Table 3:** Baseline sociodemographic and behavioural variables predicting depressive symptoms and sleep complaints at follow-up (*N* = 4848)

	Depressive symptoms			Sleep complaints		
	*β*	95% CI	*p*	OR	95% CI	*p*
Baseline depressive symptoms	*0.397*	*0.365–0.422*	*< 0.001*	*6.795*	*5.860–7.879*	*< 0.001*
Baseline sleep complaints^a^	*0.067*	*0.135–0.311*	*< 0.001*	*1.138*	*1.083–1.195*	*< 0.001*
Baseline depressive symptoms × sleep complaints interaction	*0.030*	*0.012–0.170*	*0.024*	*0.836*	*0.724–0.965*	*0.015*
Age (years)	*0.044*	*0.003–0.013*	*0.002*	0.998	0.989–1.007	0.671
Sex^b^	0.021	− 0.014–0.145	0.108	*1.380*	*1.186–1.605*	*< 0.001*
Ethnicity^c^	0.024	− 0.004–0.604	0.053	0.952	0.539–1.681	0.864
Relationship status^d^	0.015	− 0.042–0.143	0.282	*0.740*	*0.621–0.883*	*0.001*
Wealth	*− 0.028*	*− 0.062–0.000*	*0.047*	–		
1 (lowest)	–			Reference		
2				0.903	0.707–1.154	0.415
3				0.838	0.652–1.075	0.164
4				*0.768*	*0.596–0.991*	*0.042*
5 (highest)				*0.674*	*0.518–0.878*	*0.003*
Smoker^e^	*− 0.044*	*− 0.326–0.084*	*0.001*	*0.761*	*0.609–0.950*	*0.016*
Alcohol consumption	0.022	− 0.007–0.073	0.108	–		
> 5 times/week	–			Reference		
1–4 times/week				1.005	0.831–1.215	0.959
Occasionally				1.005	0.801–1.262	0.963
Rarely/never				1.137	0.899–1.437	0.283
Physical activity^f^	*− 0.039*	*− 0.212–0.044*	*0.003*	*0.830*	*0.705–0.978*	*0.026*
BMI (kg/m^2^)	*0.053*	*0.008–0.023*	*< 0.001*	*1.016*	*1.002–1.031*	*0.026*
Hypertension^g^	− 0.006	− 0.098–0.062	0.659	0.976	0.839–1.136	0.753
CHD^g^	0.014	− 0.061–0.208	0.287	1.168	0.914–1.492	0.214
Stroke^g^	0.019	− 0.051–0.368	0.137	1.146	0.785–1.672	0.480
Diabetes/high blood glucose^g^	0.019	− 0.036-0.234	0.152	1.034	0.807–1.326	0.789
Cancer^g^	0.006	− 0.136-0.221	0.640	1.125	0.810–1.562	0.483
Pulmonary disease^g^	*0.032*	*0.054–0.440*	*0.012*	1.312	0.932–1.848	0.120
Arthritis^g^	*0.050*	*0.076–0.240*	*< 0.001*	*1.363*	*1.174–1.583*	*< 0.001*

N.B. italic script denotes signifcant results at *p* < 0.05

^a^Low sleep complaints

^b^Male

^c^White

^d^Married/cohabiting

^e^Smoker

^f^Moderate/vigorous physical activity < 1 a week

^g^Not reported

### Measures

#### Depressive Symptoms and Sleep Symptoms

Depressive symptoms were measured at wave 4 and wave 6 using the 8-item Centre for Epidemiological Studies Depression scale (CES-D). The CES-D measures symptoms that can be used to identify people at risk of depression, rather than clinical depression per se [21]. The psychometric properties of the 8-item version have been shown to be comparable to the original 20-item version [22]. For the purpose of analyses described here, the sleep item was removed from the CES-D so as to avoid the issue of shared variance with the sleep problems measure. We computed a summary score by adding responses to all seven dichotomous questions (possible range 0–7). Analyses were performed using this summary score, though for descriptive purposes and post hoc tests, we also implemented the validated cut-off of 4 [19] to capture low/high depressive symptoms. The Cronbach’s alphas for the CES-D in this study were 0.796 and 0.744 (wave 4 and 6, respectively).

Sleep problems were measured with three questions referring to the most frequent insomnia symptoms, including difficulties falling asleep, waking up several times a night and waking up in the morning feeling tired. These sleep items were derived from the Jenkins Sleep Problems Scale [23]. Participants answered these questions with regard to the past month. Items were rated on a 4-point Likert scale ranging from “not during the past month” to “three or more times a week”. Scores were summed with higher scores indicating more sleep problems. The Cronbach’s alphas in this study were 0.603 and 0.585 (wave 4 and 6, respectively). Sleep duration was measured with an open-ended question asking participants about their sleep duration on an average week night. A binary variable was created using the 75th percentile on the Jenkins measure (equivalent to a score of 9/12 in our sample) and a cut-off of 5 h per night to indicate those with low/high sleep complaints. Our definition of short sleep duration as < 5 was chosen for its clinical relevance [24, 25].

#### Correlates

Correlates were all measured at baseline and were selected since they have been found to be related to both depressive symptoms [26–29] and sleep measures [18, 30–32]. Sociodemographic variables include age, sex, and whether participants were married or cohabiting with a partner. Socioeconomic status was defined as quintiles of net financial wealth, which refers to participants’ gross financial wealth with financial debt subtracted, with 1 representing the lowest wealth and 5 the highest. Height and weight were collected during the wave 4 nurse visit and BMI was derived using the standard formula (kg/m^2^). Whether or not participants reported being a current smoker (yes/no) and frequency of alcohol consumption (<twice a year/never (rarely), 1–2 times a month/every 2 months (occasionally), 1–4 times per week, >5 times per week) were also considered. Participants reported the frequency in which they engaged in vigorous, moderate, and mild physical activity and we used these data to derive two possible categories reflecting regularity of physical activity: moderate/vigorous activity once a week or less, moderate/vigorous activity more than once a week. Doctor diagnosis of hypertension was self-reported (yes/no) and these responses were combined with objective assessments taken at the nurse visit (hypertension defined as systolic blood pressure > 140 and diastolic blood pressure > 90) to generate a binary variable (yes/no). Doctor diagnosed physical illnesses were self-reported and included coronary heart disease (defined as angina and/or myocardial infarction), arthritis (defined as including both rheumatoid and osteoarthritis), pulmonary disease, cancer, and diabetes/high blood glucose.

Biomarkers were assessed from blood drawn from participants’ forearm during the nurse visit and included high sensitivity C-reactive protein (hs-CRP) and fibrinogen. Participants who had a clotting or bleeding disorder and those on anti-coagulant medication did not provide blood samples. Fasting samples, defined as not eating or drinking for 5 h prior to blood draw, were collected where possible and when not otherwise contraindicated (e.g. > 80 years, diabetic, frail or unwell, ever had a seizure). Hs-CRP was measured using the N Latex CRP mono immunoassay on the Behring Nephelometer II analyser. Fibrinogen was analysed using a modification of the Clauss thrombin clotting method on the Organon Teknika MDA 180 analyser. All blood samples were analysed at the Royal Victoria Infirmary laboratory in Newcastle upon Tyne, UK (for a detailed description of blood analyses see [33]).

### Statistical Analysis

Data were assessed for assumptions of parametric data prior to analysis. Extreme outliers greater than 3 standard deviations from the mean were removed from the fibrinogen and hs-CRP data and since the hs-CRP data were positively skewed, logarithmic transformation was performed to normalise the distribution. Associations between depressive symptoms and sleep complaints and the potential correlates were tested using Pearson’s correlations, *t* tests, ANOVAs, and chi-square tests, as appropriate. Linear regression and logistic regression analyses were performed to assess the prospective association between the sociodemographic, behavioural, and clinical variables measured at wave 4 and both depressive symptoms and sleep complaints measured at wave 6 as appropriate. Predictor variables were entered into models simultaneously. These analyses also controlled for baseline depressive symptoms and sleep complaints to explore the direction of the association between depression and poor sleep. Importantly, we included a mean-centred interaction term in these models for baseline depressive symptoms × baseline sleep complaints. Separate analyses were performed for depressive symptoms and sleep complaints. Results are presented as standardised coefficients (Beta) for linear regression and odds ratios for logistic regression with 95% confidence intervals (CI). We have presented the models excluding fibrinogen and hs-CRP to maximise the sample size, though analyses including biomarkers are reported in the text. Finally, post hoc tests were performed to examine the significant interaction results in greater detail using fully adjusted linear and logistic regression models as appropriate. Specifically, we split the dataset by low/high depressive symptoms (CES-D ≥ 4) to test the effect of baseline sleep complaints on follow-up depressive symptoms at wave 6, and for models using baseline depressive symptoms to predict wave 6 sleep complaints we split the file by wave 4 low/high sleep complaints. Data were analysed using SPSS version 21. Exact *p* values are reported throughout.

## Results

The characteristics of the sample are displayed in Table 1. The mean age of the sample was 65.71 years (± 8.76 years) and the majority were of White ethnic origin and married or cohabiting with a partner. Most participants did not smoke, but only a third engaged in moderate or vigorous activity at least once a week. The mean BMI of 28.35 kg/m^2^ (± 5.20 kg/m^2^) is reflective of the high levels of obesity in the sample; 30.2% of participants had a BMI ≥ 30. Physical illness was prevalent in the sample, with almost half of participants reporting hypertension and over a third reporting arthritis. Mean depressive symptom scores at baseline (0.90 ± 1.56) were overall low; however, 9% of participants scored above the threshold of 4 for the CES-D indicating possible depression. Interestingly, of those with high levels of depressive symptoms (CES-D ≥ 4), very few reported low sleep complaints (131/460, 3.7%). This reflects the high comorbidity between depressive symptoms and poor sleep using our cut-offs in our sample (*χ* ^2^ = 397.44, *p* < 0.001).

### Associations Between Depressive Symptoms and Sleep Complaints and Demographic, Behavioural and Biological Correlates at Wave 4

Table 2 shows the results from the univariate analyses comparing depressive symptom scores on the CES-D and those with and without high sleep complaints across a wide range of potential correlates at wave 4. The results show that apart from age, ethnicity, stroke, and cancer, higher depressive symptoms and high sleep complaints were similarly associated to sociodemographic, behavioural, clinical, and biological variables, compared to those lower in these respective symptoms.

Specifically, greater depressive symptoms and high sleep complaints were both associated with female sex, non-cohabitation, having lower wealth, smoking, engaging in less physical activity, consuming alcohol less frequently, having a higher BMI, reporting a diagnosis of hypertension, CHD, diabetes/high blood glucose, pulmonary disease, or arthritis and having higher circulating levels of fibrinogen and hs-CRP (all *p* < 0.05). Older age (*r* = 0.048, *p* = 0.001) and non-White ethnicity (*t* = − 2.335, *p* = 0.020) were associated with higher depressive symptoms, as were reporting a positive diagnosis of stroke (*t* = − 2.016, *p* = 0.044) and cancer (*t* = − 4.022, *p* < 0.001); however, congruent associations were not observed for low/high sleep complaints.

### Predicting Depressive Symptoms and Sleep Complaints at Wave 6

Regression analyses were performed to assess the prospective association between the sociodemographic, behavioural, and clinical variables measured at wave 4 and both depressive symptoms (linear regression) and sleep complaints (logistic regression) measured at wave 6. Results for these analyses are displayed in Table 3. Fibrinogen and hs-CRP were both non-significant predictors and were thus removed from the models to maximise the sample size (*N* = 4848). Specifically, when the biomarkers were included in the model (*N* = 3579), both fibrinogen and hs-CRP were non-significant predictors of depressive symptoms (fibrinogen: *β* = − 0.022, 95% CI 0.164–0.035, *p* = 0.207; hs-CRP: *β* = 0.015, 95% CI − 0.030–0.073, *p* = 0.406) and sleep complaints (fibrinogen: OR 0.983, 95% CI 0.812–1.190, *p* = 0.860; hs-CRP: OR 1.045, 95% CI 0.946–1.154, *p* = 0.391) at follow-up.

Analyses predicting wave 6 depressive symptoms showed significant predictors to include greater baseline depressive symptoms (*β* = 0.397, 95% CI 0.365–0.422), high baseline sleep complaints (*β* = 0.067, 95% CI 0.135–0.311), older age (*β* = 0.044, 95% CI 0.003–0.013), higher BMI (*β* = 0.053, 95% CI 0.008–0.023), pulmonary disease (*β* = 0.032, 95% CI 0.054–0.440), and arthritis (*β* = 0.050, 95% CI 0.076–0.240). Negative associations were also found, with lower wealth (*β* = − 0.028, 95% CI − 0.062–0.000), being a non-smoker (*β* = − 0.044, 95% CI − 0.326 −0.084) and not engaging in regular moderate/vigorous physical activity (*β* = − 0.039, 95% CI 0.212 −0.044) being associated with a greater depressive symptoms 4 years later. Interestingly, we also found a significant depressive symptoms × sleep complaints interaction (*β* = 0.030, 95% CI 0.012–0.170). We performed a post hoc fully adjusted linear regression model to examine this interaction further. Results showed that baseline sleep complaints were a significant predictor of greater depressive symptoms at wave 6 follow-up only among those participants with low (i.e. CES-D < 4) baseline depressive symptoms (*β* = 0.128, 95% CI 0.290–0.462, *p* < 0.001) and not high (i.e. CES-D ≥ 4) (*β* = 0.063, 95% CI − 0.188–0.829, *p* = 0.217).

Analyses predicting the increased odds of sleep complaints at wave 6 showed baseline depressive symptoms (OR 6.795, 95% CI 5.860–7.879) and sleep complaints (OR 1.138, 95% CI 1.083–1.195) to be significant predictors. Female sex (OR 1.380, 95% CI 1.186–1.605), higher BMI (OR 1.016, 95% CI 1.002–1.031), and having arthritis (OR 1.363, 95% CI 1.174–1.583) were also associated with increased odds of elevated sleep complaints 4 years later. Relative wealth (e.g. the highest wealth quintile (5) compared to the lowest [1] OR 0.761, 95% CI 0.609–0.950), cohabitation (OR 0.740, 95% CI 0.621–0.883), and regular physical activity (OR 0.830, 95% CI 0.750–0.978) were associated with a reduction in the odds of sleep complaints at wave 6. We also found a significant depressive symptoms × sleep complaints interaction (OR 0.836, 95% CI 0.724–0.965). We performed a post hoc fully adjusted logistic regression model to examine this interaction further. Results showed that baseline depressive symptoms were a significant predictor of high sleep complaints at wave 6 follow-up only among those participants with low baseline sleep complaints (OR 1.263, 95% CI 1.176–1.356, *p* < 0.001) and not high (OR 1.034, 95% CI 0.975–1.096, *p* = 0.268).

## Discussion

This study aimed to characterise the similarities and differences in adults with depressive symptoms and sleep complaints in order to better understand the common comorbidity between the two. We investigated the sociodemographic, behavioural, clinical and biological correlates of depressive symptoms and sleep complaints using data from ELSA, a nationally representative cohort of adults aged 50 years and older living in England. Our findings showed that older men and women reporting elevated depressive symptoms and/or sleep complaints shared a range of sociodemographic, behavioural and clinical correlates that included female sex, non-cohabitation, lower wealth, smoking, exercising less than once a week, drinking alcohol infrequently, higher BMI, having hypertension, CHD, diabetes/high blood glucose, pulmonary disease, and arthritis, as well as higher levels of hs-CRP and fibrinogen. When explored prospectively, elevated levels of depressive symptoms and sleep complaints also shared common risk factors, namely, baseline depressive symptoms, sleep complaints, relative poverty, smoking, physical inactivity, higher BMI, and arthritis. Interestingly, an interaction effect was shown between baseline depressive symptoms and sleep complaints such that high sleep complaints at wave 4 predicted greater depressive symptoms at wave 6 only among those with low depressive symptoms at baseline, and greater depressive symptoms at wave 4 predicted high sleep complaints at wave 6 only in those with low sleep complaints at baseline.

We found a high comorbidity between depressive symptoms and poor sleep using the cut-offs employed in our study since the vast majority (96.7%) of those reporting elevated depressive symptoms also suffered from disturbed and/or short sleep. While previous studies have reported that approximately 50–60% of depressed patients have clinically defined insomnia; in epidemiological studies, results are consistent with our findings, with reports that 83% of depressed individuals have at least one insomnia symptom, with this figure rising to 90% among depressed 55–64 year olds [34].

Our cross-sectional findings at baseline identified that older adults with elevated depressive symptoms and sleep complaints had similar sociodemographic, economic, behavioural, and clinical correlates. Our study is the first, to the best of our knowledge, to draw a parallel between these two separate literatures. The evidence for the detrimental effect of unhealthy lifestyles and negative health behaviours on sleep and depression is well established [35–37]. Socioeconomic status was also identified in our analyses as an important correlate of depressive symptoms and sleep complaints, and again, this finding is congruent with the literature [30, 38], with the most economically deprived individuals being at greatest risk of poor mental and physical health. Our findings on the association between depressive symptoms and sleep complaints and cohabitation are also in line with past research showing marriage has largely positive effects on depressive symptoms [39] and sleep [40]. Interestingly, we found evidence that greater depressive symptoms and sleep complaints were associated with low alcohol consumption; this is largely inconsistent with previous literature [41, 42], although health benefits of moderate alcohol consumption, defined as 1 to 3 alcoholic drinks per day, have been reported for both outcomes [32, 43]. In our analyses, frequency of alcohol consumption was measured on a scale from more than five times a week to rarely or never; as such, our highest frequency category may overlap with the definition of moderate drinking provided by Ferrerira and Weems [43]. More research is needed to corroborate whether moderate drinking on a regular basis confers depression and sleep benefits.

Both depressive symptoms and high sleep complaints were associated with a range of negative health factors, including cardiovascular diseases (i.e. hypertension, stroke, CHD), arthritis, pulmonary disease, higher BMI, and higher levels of fibrinogen, and hs-CRP. This corroborates research suggesting both depression [27, 44] and poor sleep [45] are associated with higher levels of inflammation. Obesity is known to be associated with higher levels of inflammation [46] as are physical illnesses, potentially contributing to these findings.

Our prospective findings only partly supported our cross-sectional analyses. Specifically, the only baseline factors that predicted both sleep complaints and depressive symptoms at follow-up were poor sleep, greater depressive symptoms, relative poverty, higher BMI, smoking, physical inactivity, and arthritis. In individual models, elevated depressive symptoms were predicted by baseline depressive symptoms and sleep complaints, older age, higher BMI, pulmonary disease and arthritis, while not smoking, engaging in regular physical activity and higher wealth were protective factors. As previously discussed, smoking, physical inactivity and low socioeconomic status are known to be associated with depression [36, 38, 47]. Our data suggest these factors may precede depression in the temporal pathway. In terms of increased future risk of sleep complaints, we found that female sex, higher BMI and arthritis were all significant predictors, while not smoking, engaging in regular physical activity, cohabitation and higher wealth were protective factors. It has been long recognised that women are more likely to report poor sleep [30]. Meta-analytic reviews suggest that poor and short sleep increase the risk of obesity and chronic physical conditions [18] but the link between poor sleep and physical health is bi-directional [48], and it is plausible that excess weight and arthritis, which is often a source of considerable pain, may seriously disturb the ability to sleep [49, 50]. The association between pain and arthritis has also been suggested as a reason why rheumatoid arthritis is often associated with depression [51]; this may partly explain the association we found here between arthritis and depression.

The reason why the other sociodemographic, behavioural, clinical, and biological factors were not significant in prospective models is interesting and informs our understanding of the causal associations between these factors. Particularly notable is the lack of prospective association found between the clinical and biological factors and future depressive symptoms and sleep complaints. Previous research has supported the association between physical illness and future depression [52] and inflammatory markers have been shown to be associated with disrupted sleep, particularly in clinical populations [53]. We can only speculate why in our data physical illness (except pulmonary disease and arthritis), hs-CRP and fibrinogen did not predict future sleep complaints and depressive symptoms. It is plausible that inflammatory factors may impact sleep and depression when more severe or persistent levels of inflammation, sleep or depressive symptoms are present [54], as reported in those with clinically diagnosed depression [53, 55]. Moreover, we were unable to take into account disease stage, disease severity, and treatment of diseases that could possibly mask symptoms, which may all impact the prospective association with depressive symptoms and sleep complaints. Finally, since sleep data were only introduced in ELSA at wave 4, our follow-up analysis was restricted to 4 years which may preclude a significant association from being detected.

We found that future high sleep complaints were predicted by baseline depressive symptoms only among those with low sleep complaints at baseline. Moreover, future depressive symptoms were only predicted by sleep complaints in those with low depressive symptoms at baseline. These results require careful interpretation given there were few participants with high depression symptoms and low sleep complaints in our sample. However, they do add to a growing literature on the causal sequence between sleep and depression. Our findings could be considered to partly support the work of Skapinakis and colleagues [19] who suggest that their results of a strong cross-sectional association between poor sleep and depression, but lack of prospective association, perhaps points to sleep complaints being a prodromal or residual symptom of depression. Future work is needed to understand these effects in greater detail. However, our findings suggest the importance of taking into account the interaction between sleep and depression symptoms in understanding these longitudinal effects and that sleep complaints and depressive symptoms both require targeted interventions and treatment. Indeed, combining standard antidepressant treatment with cognitive behavioural therapy for insomnia in patients with major depression disorder has been found to be associated with faster and more durable remission from depression than treatment with antidepressants alone [56]. There is also evidence that improving sleep quality in older adults with insomnia is associated with a reduction in the biological markers of disease including inflammatory markers [57]. This is important given the close link between inflammation and depression [44].

Our study has a number of strengths. We used a large and well-described cohort of community-dwelling older men and women from England [20]. The results described here include prospective analyses with participants being followed for 4 years to shed light on the risk factors for future depressive symptoms and sleep complaints. Because a large number of data are collected as part of ELSA, which includes information on psychological and socioeconomic factors, leisure activities, health behaviours and physical health indicators, this reduces the possibility that participants were aware of our interests in depressive symptoms and sleep described here. However, our conclusions are tempered by a number of limitations that need to be borne in mind. First of all, sleep was measured with self-report that is prone to affect and memory biases [58] as well as inaccuracies when compared with objective sleep indicators. The Jenkins sleep scale showed low internal consistency in our sample, though this in line with the original validation data for this questionnaire [20] suggesting there is a need for future scale development in this field. We do not have information on sleep disorders such as sleep apnoea or sleep medication since these are not assessed in ELSA. Similarly, depressive symptoms were assessed using the CES-D and therefore our observations are limited to elevations in depressive symptoms and not clinical depression. Finally, although self-report measures of physical illnesses have been shown to be reliable in previous cohort studies [59], to circumvent possible measurement error, we grouped together osteoarthritis and rheumatoid arthritis cases; therefore we are unable to tease apart the effects of these two distinct illnesses.

In conclusion, our findings showed that older men and women reporting elevated depressive symptoms and/or sleep complaints shared a range of sociodemographic, behavioural, biological and clinical correlates. Prospective analyses revealed that the only common risk factors for elevated depressive symptoms and sleep complaints 4 years later were elevated baseline depressive symptoms and sleep complaints, smoking, physical inactivity, BMI, arthritis and wealth. Collectively, our findings highlight the common comorbidity between depressive symptoms and sleep complaints which underscores the need for further research to understand their combined detrimental effects on long-term health and wellbeing.
